# AlN/InAlN thin-film transistors fabricated on glass substrates at room temperature

**DOI:** 10.1038/s41598-019-42822-6

**Published:** 2019-04-18

**Authors:** Kyohei Nakamura, Atsushi Kobayashi, Kohei Ueno, Jitsuo Ohta, Hiroshi Fujioka

**Affiliations:** 1 Institute of Industrial Science, The University of Tokyo, 4-6-1 Komaba, Meguro-ku, Tokyo, 153-8505 Japan; 2 ACCEL, Japan Science and Technology Agency, 7 Gobancho, Chiyoda-ku, Tokyo, 102-0076 Japan

**Keywords:** Materials for devices, Electronic devices

## Abstract

In this study, InAlN was grown on glass substrates using pulsed sputtering deposition (PSD) at room temperature (RT) and was applied to thin-film transistors (TFTs). The surface flatness of the InAIN films was improved by reducing the growth temperature from 350 °C to RT. Further, the electron mobility and concentration of the InAlN film that was grown at RT were observed to be strongly dependent on the In composition. It was also observed that the electron concentration could be reduced during the introduction of Al atoms into InN, which could most likely be attributed to the reduction in the position of the Fermi level stabilization energy with respect to the conduction band edge. Further, InAlN-TFT was fabricated, and successful operation with a field-effect mobility of 8 cm^2^ V^−1^ s^−1^ was confirmed. This was the first demonstration of the operation of TFTs based on the growth of InAlN on an amorphous substrate at RT.

## Introduction

Thin-film transistors (TFTs), which are field-effect transistors that are fabricated on insulating substrates, such as glass or polymers, are indispensable electronic elements for display electronics and other applications^1^. The TFT performance is primarily dependent on the electron mobility of the channel layer of a given TFT. Amorphous Si or oxide semiconductors, such as InGaZnO, are generally used as TFT channel materials in large area applications^2^. However, the field-effect mobilities of these TFTs are limited. A new channel material with a high electron mobility could be used for developing high-performance micro-light-emitting diode displays, which require high-density current for operation.

Group-III nitrides have been already used as transistors for developing high-speed communication systems^3,4^. They may also be employed for developing high-performance next-generation TFTs if they are successfully grown on glass or polymer substrates. Because InN exhibits the highest electron mobility and velocity among group-III nitrides^5,6^, it is considered to be a good TFT material candidate.

It is well known that metal-organic chemical vapor deposition (MOCVD), which is commonly used for the mass production of group-III nitride devices, requires a high growth temperature to decompose ammonia and metal-organic compound gas as well as to initiate nitride crystal growth^7^. However, the high growth temperature of MOCVD is incompatible with glass or polymer substrates. Recently, it has been demonstrated that the use of pulsed sputtering deposition (PSD) helps to considerably reduce the growth temperature of group-III nitrides while maintaining excellent optical and electrical qualities^8–12^. In fact, the successful fabrication and operation of InN-based TFTs on glass substrates or polyimide sheets using PSD were demonstrated^13,14^. However, the sheet electron density of InN was considerably high even with the small thickness, thereby making it difficult to obtain the off state even under high negative gate biases. This difficulty originates from the Fermi level of the InN film being pinned in the conduction band at the surface and the back interface because of the formation of a high density of defects^15^, thereby causing InN to strongly tend to become a degenerate n-type semiconductor. This problem may be solved by adding Al atoms to the InN channel layer. It is reasonable to assume that the Fermi level stabilization energy can be pushed into the forbidden gap by changing InN to InAlN^16^. Because the bandgap of InAlN is considerably larger than that of InN, the high-density electrons at the top and bottom interfaces of the channel layer, which degrade the TFT performance, should be reduced.

In this study, we investigated the crystallinity, surface morphology, and electrical properties of the InAlN films grown on glass substrates at room temperature (RT) by PSD and the TFTs that were fabricated using the InAlN films grown at RT.

**Figure 1:**
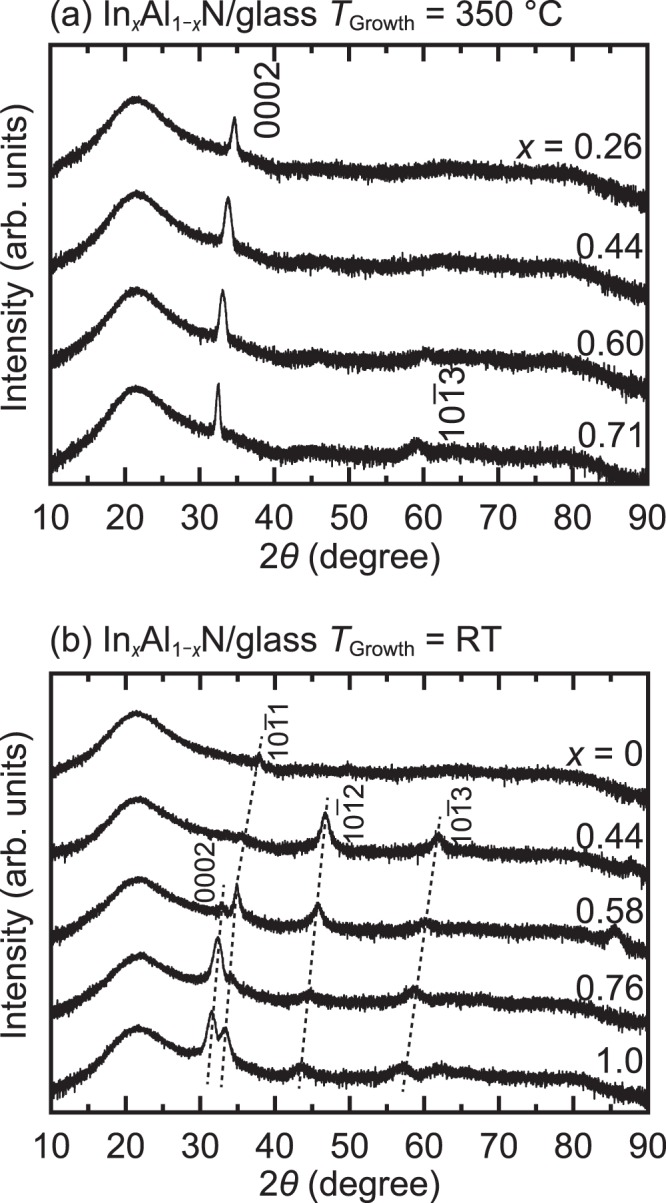
The XRD patterns of InAlN films grown on glass substrates at (**a**) 350 °C and (**b**) RT.

The crystallinities of the InAlN films that were grown on glass substrates were investigated using XRD. Figure 1(a) depicts the XRD patterns of the InAlN films with various In compositions that are grown at 350 °C on glass substrates. The In compositions were observed to be 0.26, 0.44, 0.60, and 0.71 from top to bottom in the figure. Regardless of the In composition, the 0002 peaks were observed with strong intensities, indicating the growth of highly *c*-axis-oriented polycrystalline InAlN films. In addition to the 0002 peak, a $10\bar{1}3$ peak was also observed for In_0.71_Al_0.29_N; however, it gradually disappeared as the In content decreased, indicating that the orientation tendency of *c*-axis was enhanced with decreasing In content. These phenomena can probably be explained by the tendency of AlN to exhibit its *c*-plane on the surface at this growth temperature.

Further, the crystallinities of InAlN films that were grown at RT were also studied using XRD. Figure 1(b) depicts the XRD patterns of the InAlN films with In compositions of 0 (AlN), 0.44, 0.58, 0.76, and 1 (InN) from top to bottom. As the In composition decreased, the 0002 peak disappeared, and other peaks, derived from $10\bar{1}1$ or $10\bar{1}2$, were observed. Unlike the InAlN film that was grown at 350 °C, the crystallinity of the InAlN film that was grown at RT degraded with decreasing In content (approaching AlN). This result indicates that an In-rich InAlN film is preferable for fabricating TFT at RT.

**Figure 2:**
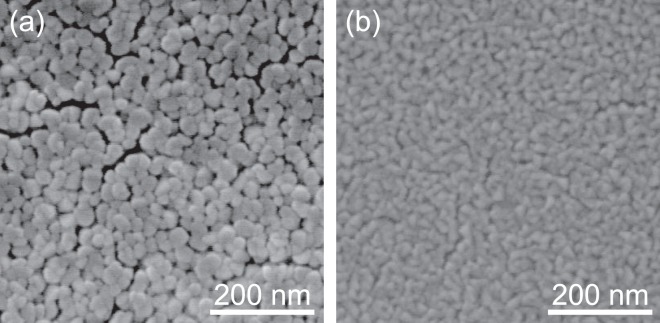
The SEM images of InAlN films grown at (**a**) 350 °C and (**b**) RT.

Figure 2 compares the SEM surface images of In_0.75_Al_0.25_N films that are grown on glass substrates at (a) 350 °C and (b) RT. The grain size of the InAlN film that was grown at 350 °C was observed to be approximately 40 nm on average, while that of the InAlN film that was grown at RT was observed to be approximately 20 nm on average. The number of grains per unit area (grain density) of the InAlN film that was grown at RT was approximately 2.5 times larger than that of the InAlN film that was grown at 350 °C. This is probably because the surface migration of the film precursor was enhanced more significantly at 350 °C as compared to that at RT. Although the size of the grains in RT-InAlN was relatively small, its surface was sufficiently smooth for it to be employed as a channel layer for a field-effect transistor.

**Figure 3:**
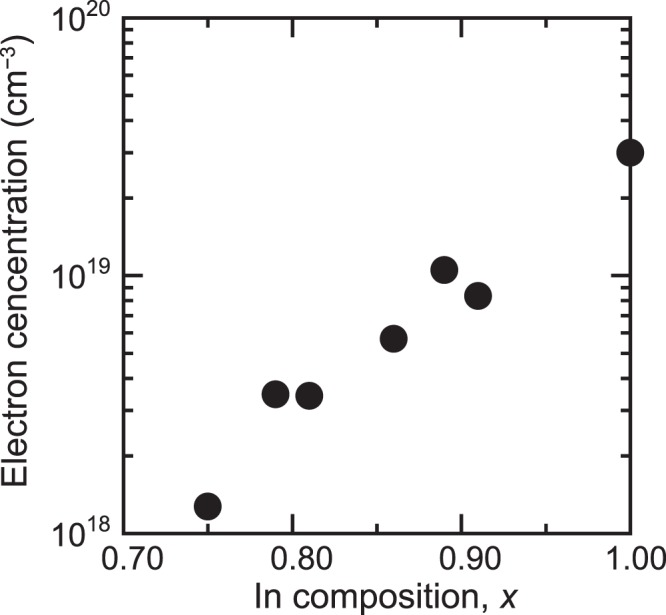
The electron concentration of the polycrystalline InAlN films grown at RT.

**Figure 4:**
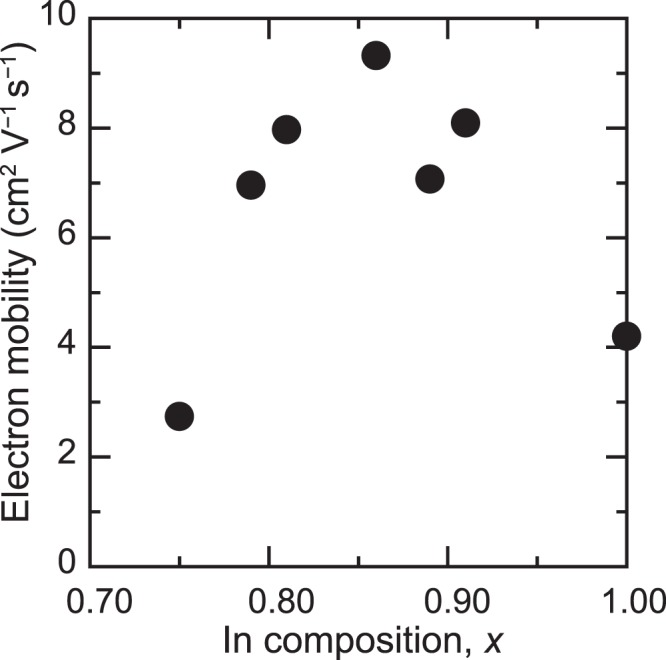
The electron mobility of the polycrystalline InAlN films grown at RT.

The electron concentration of ~150-nm-thick InAlN films that were grown at RT on glass substrates was investigated using the Hall-effect measurements. As can be observed from Fig. 3, the decreasing In content causes the monotonical decrease of the electron concentration of InAlN. This can be probably attributed to the reduction in the position of the Fermi level stabilization energy with respect to the conduction band edge. In addition, as can be observed from Fig. 4, the electron mobilities of the films increased with the addition of Al atoms to InN and reached maximum mobility when the Al content became 0.14. These results indicate that the addition of Al atoms to the poly InN is considerably promising for preparing nitride TFTs on amorphous substrates at RT.

**Figure 5:**
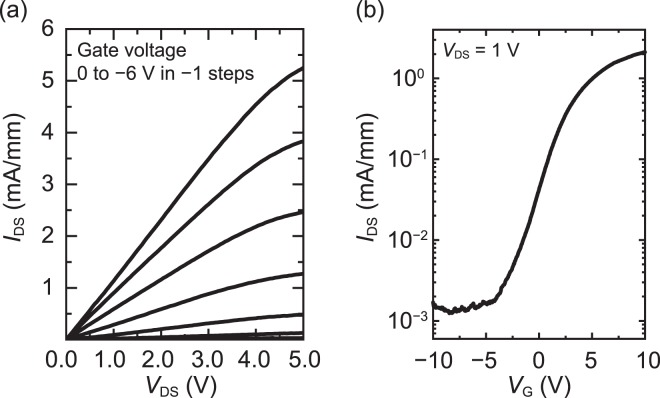
(**a**) The output and (**b**) transfer characteristics of In_0.79_Al_0.21_N-TFT.

Finally, TFTs were fabricated using the InAlN film that was grown at RT to form a channel layer. Further, the gate dielectrics of AlN that was deposited at RT by PSD were employed. Figures 5(a,b) depict the output and transfer characteristic of an InAlN-TFT with a channel thickness of approximately 7 nm, respectively. The In composition of the channel layer was observed to be 0.79. An estimated bandgap of the In_0.79_Al_0.21_N is 0.82 eV.The TFT was operated in the n-type depletion mode, which indicated that the InAlN film that was grown at RT exhibited an electron conduction channel even at a gate bias of zero volts. The drain current clearly decreases upon the application of negative gate bias to the TFT. This is the first demonstration of transistor operation using the InAlN film that was grown at RT on amorphous substrates. Further, the field effect mobility (μ_*FE*_) of the device was calculated using the following formula:

$${\mu }_{FE}=\frac{G{m}_{max}\times L}{{V}_{DS}\times {c}_{i}\times W},$$

where *Gm*_*max*_, *L*, *V*_*DS*_, *c*_*i*_, and *W* denote the maximum transconductance, channel length, drain voltage, capacitance of the gate dielectric, and channel width, respectively. The calculated ${\mu }_{{FE}}$ of the TFT with a *W* of 50 μm and an *L* of 5 μm was 8 cm^2^ V^−1^ s^−1^. The on-to-off ratio of the drain current was observed to be ~10^2^. The transconductance and field-effect mobility were calculated without considering the parasitic resistance, which means that the field-effect mobility was slightly underestimated. Although characterization of electrical properties of InAlN with In composition of ~0.8 films is not found in the literatures, the present mobility of polycrystalline In_0.79_Al_0.21_N is much lower than those of single-crystal InN films^17^. It should be noted that the field effect mobility of 7-nm-thick InAlN TFT is comparable to the Hall mobility of the 150-nm-thick InAlN. Shao *et al*. reported that the field effect mobility of a 7-nm-thick a-IGZO TFT was almost same as that of thicker (~30 nm) a-IGZO TFTs^18^. It was also reported that electron transport in a-IGZO is governed by percolation conduction. These findings indicate that the conduction mechanism in InAlN grown at RT is possibly similar to that in a-IGZO. Microstructures of the InAlN films are currently under investigation with transmission electron microscopy. We have also observed hysteresis of 0–5 V in the transfer curves, but we have not identified the origin of the hysteresis yet. We speculate that the hysteresis comes from trapping of electrons at grain boundaries of InAlN or interfaces of AlN/InAlN. Further improvement of morphology of polycrystalline InAlN is necessary to achieve the device operation without the hysteresis in the transfer curves.

In this study, InAlN films were grown on synthetic quartz glass substrates at low temperatures, and their characteristics were evaluated. The XRD measurements revealed that the *c*-axis orientation tendency of the InAlN films depended on the growth temperature. By reducing the growth temperature to RT, the surface flatness of the InAlN film was improved. It was also observed that the introduction of Al atoms in the InN film could reduce the electron concentration, which was most likely to be caused because of a reduction in the position of the Fermi level stabilization energy with respect to the conduction band edge. TFTs were fabricated using the InAlN layers that were grown at RT. The TFTs operated with a field-effect mobility of 8 cm^2^ V^−1^ s^−1^. These results indicate that RT-InAlN is considerably promising for the preparation of transistors on thermally vulnerable substrates such as glass or polymers.

## Method

The InAlN films were grown on synthetic quartz glass substrates at RT and 350 °C by PSD. The InAlN film growth was performed under a flow of purified nitrogen gas (99.9999% purity) and argon gas. The growth temperature was determined from a thermocouple that was located near the substrates. Further, the structural and electrical properties of the films were investigated by X-ray diffraction (XRD) (Rigaku RINT 2500V), scanning electron microscope (SEM) (JEOL JSM-6500F) observation, and Hall-effect measurements (Toyo ResiTest 8400AC) using the van der Pauw method. TFTs were fabricated using photolithography and lift-off techniques. The Ti (30 nm)/Al (60 nm) layers were deposited as the source and drain electrodes of the TFTs by electron beam evaporation, which was followed by the formation of an AlN gate dielectric layer (35 nm) by PSD at RT. The device fabrication was finalized with the deposition of the Au gate electrode (100 nm) by electron beam evaporation. The TFT exhibited a top gate structure, and the electrical characteristics of the TFTs were evaluated using a semiconductor parameter analyzer (Keysight 4155C). For the calculation of the capacitance of AlN gate, we used the dielectric constant of AlN (7.2), which was deduced from *C*–*V* measurements (Keysight 4284A) of room-temperature-deposited AlN (thickness of 96 nm, electrode diameter of 500 μm).
